# Recycled Aggregate Integration for Enhanced Performance of Polymer Concrete

**DOI:** 10.3390/ma17164007

**Published:** 2024-08-12

**Authors:** Maja Kępniak, Filip Chyliński, Paweł Łukowski, Piotr Woyciechowski

**Affiliations:** 1 Department of Building Materials Engineering, Faculty of Civil Engineering, Warsaw University of Technology, Armii Ludowej 16, 00-637 Warsaw, Poland; pawel.lukowski@pw.edu.pl (P.Ł.); piotr.woyciechowski@pw.edu.pl (P.W.); 2 Instytut Techniki Budowlanej, Filtrowa 1, 00-611 Warsaw, Poland; f.chylinski@itb.pl

**Keywords:** recycled aggregate, eco-friendly concrete, polymer concrete, polymer composites, sustainability

## Abstract

The objective of the research outlined in this paper is to propose an eco-friendly solution that simultaneously contributes to improving the characteristics of polymer composites. The analyzed solution entails the use of recycled aggregate from crushed concrete rubble. The authors conducted experiments to test the consistency, density, flexural strength, compressive strength, and microstructure of polymer concrete (PC) with different proportions of recycled aggregate (RA). It was found that PC with RA had a higher compressive strength, 96 MPa, than PC with natural aggregate, 89.1 MPa, owing to the formation of a double-layer shell of resin and calcium filler on the surface of porous RA grains. Using a resin with a lower viscosity could improve the performance of PC with RA by filling the cracks and penetrating deeper into the pores. RA is a valuable material for PC production, especially when it contains porous grains with poor mechanical properties, which are otherwise unsuitable for other applications. This article also highlights the environmental and economic benefits of using RA in PC, as it can reduce waste generation and natural resource consumption.

## 1. Introduction

The construction sector is facing increased pressure to become more sustainable in recent times because of stricter regulations on waste and the need for renewable chemicals and fuels. This can help reduce costs and satisfy customers’ expectations. One effective way to achieve this is a circular economy, which can promote a long-term sustainable construction industry [1,2,3,4]. The International Solid Waste Association reports that the world produces approximately 4 billion tons of waste every year, and this number is expected to increase by 2025 [5]. Approximately half of this is municipal waste, while the rest comes from industrial and production activities, which are growing in both developed and developing countries because of population, living standards, and urbanization growth. There are also large amounts of construction and demolition waste generated every year, which are dumped in landfills. This problem as well as the scarcity of natural resources have motivated the search for innovative solutions that can support a circular economy. Reusing recycled cement concrete aggregate as a new aggregate to produce building composites can help solve the waste problem and conserve natural aggregate resources. Many researchers have shown that recycled aggregate can be a good alternative aggregate for producing concrete, both in terms of environmental and economic benefits, as well as considering life cycle analysis (LCA) [6,7,8]. It should also be noted that polymer concrete is widely used in the building industry as a composite with a low carbon footprint compared to cement concrete. Polymer concrete has many advantages such as easy preparation, rapid hardening, good abrasion, high strength, good corrosion, and frost resistance [9,10,11,12,13]. Polymer concrete differs from cement concrete in terms of mechanical properties, as it exhibits a significantly higher compressive strength, approximately 2–3 times greater than that of regular concrete, and up to 5 times higher flexural and tensile strengths. This makes it a material that is less prone to cracking. Additionally, these strengths are typically achieved within about a day, which is a substantial reduction in curing time compared to conventional concrete. There is usually no reaction between the polymer matrix and aggregate particles [14]. Therefore, the substitution of natural aggregates with recycled aggregates in the production of polymer concrete is an effective method for waste material management that preserves natural resources. Many studies have been conducted on the effective use of waste dust materials [15,16,17] and plastic waste [17,18] as ingredients in polymer concrete. The use of recycled cementitious aggregates for the production of cement concrete is limited by the carbonation that occurs [19,20], which can lead to the corrosion of reinforcing steel. This problem does not occur with polymer concrete. Due to their significant flexural and tensile strengths, polymer concretes do not require steel reinforcement. In contrast, steel reinforcement in cement concrete needs protection provided by low pH levels, which limits the use of carbonated recycled aggregate particles in cement concrete [21]. The use of concrete construction and demolition waste (CDW) aggregates to produce polymer concrete could be economically and environmentally beneficial. In this context, the productive use of waste can be a way to reduce some of the challenges related to their management, decrease dependence on natural resources, and, in some cases, produce environmentally friendly products. In this study, an attempt was made to replace part of the coarse aggregate with recycled aggregate, which mainly consists of crushed cement concrete. In the face of climate change and a growing focus on a circular economy, numerous studies have been conducted on the development of more sustainable building composites. Concurrently, polymer materials are widely used in the production of various sustainable construction composites. For instance, some researchers have demonstrated the beneficial impact of impregnating recycled aggregate before using it in cement concrete [22,23,24,25]. This pre-treatment aims to improve the strength of the mortar within the recycled aggregate particles. The quality of cement concrete with recycled aggregate depends on the parent material and the condition of the old concrete, which affects its application in new concrete [26]. Experiments indicate that polymeric treatment significantly enhances the compressive strength and water absorption capacity of cement concrete [26]. Additionally, incorporating polymer pre-treated with mixed recycled aggregate in concrete improves durability by reducing water permeability, sorptivity, and chloride penetration [27]. Challenges in using recycled aggregate include fluctuations in composition and their impact on concrete properties; however, the polymer-based treatment is proposed as an effective solution [28,29]. Given that pre-treatment with polymer in cement concrete is such a promising solution, it can be inferred that the collaboration between polymer and recycled aggregates is also promising. Other studies focus on the use of polymer fibers, which enhance the tensile and flexural strengths of the composites [30,31]. There are also studies focusing on modifying mineral composites with polymers as co-binders to improve the strength and durability of the composites [24,32].

The concept of designing and testing these composites primarily revolves around maintaining or even enhancing their existing properties. Polymer concrete stands out as a particularly promising material, boasting excellent strength and durability parameters, albeit at a higher cost than conventional cement composites. Consequently, despite ongoing efforts to enhance its ecological footprint, these advancements have seldom translated into production practices. The objective of the research outlined in this study was to propose an eco-friendly solution that simultaneously contributes to improving the characteristics of polymer composites. The analyzed solution entails the use of recycled aggregate from crushed concrete rubble. This study aims to determine whether substituting natural aggregate with crushed concrete aggregate can significantly enhance the strength of polymer concrete.

In the experiments, coarse aggregate of fraction 4/8 (grain size between 4 and 8 mm) was replaced by recycled aggregate in different proportions from 25 to 100%, and the consistency, density, flexural strength, compressive strength, and microstructure of the resulting composites were tested. Statistical methods were used to analyze the results. The aim of this study was to verify whether natural aggregates can be replaced by recycled aggregates in polymer composites without deteriorating their basic parameters.

## 2. Materials and Methods

**Figure 1:**
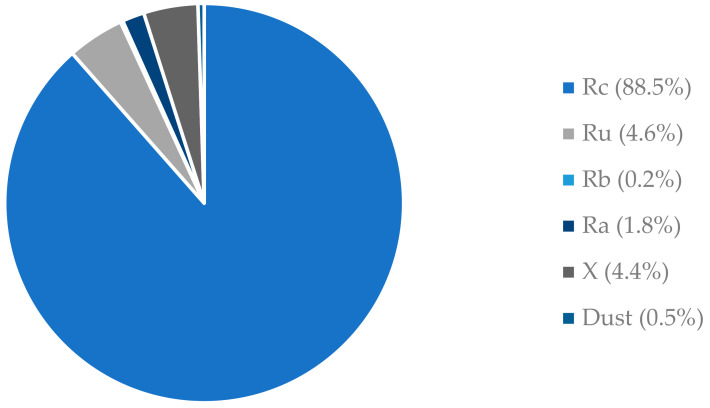
Composition of recycled coarse aggregate according to EN 933-1 [38] (percentage by weight).

The polymer used to prepare all the composites in this study was a synthetic vinyl-ester resin with low viscosity (350 ± 50 mPa·s at 25 °C) and high flexural strength and tensile strength (reported by the manufacturer as 110 MPa and 75 MPa, respectively) [33]. Therefore, concretes made from this resin should maintain high mechanical strength for long-term use, even when exposed to harsh environments. Two-size fractions of natural coarse aggregates were used: one with an aggregate size of 2–4 mm (2–4 N) [34] and the other with 4–8 mm (4–8 N) [34] with fineness moduli of 5.56 and 5.63, respectively. Both were natural gravel. Moreover, recycled coarse aggregates obtained from the demolition of concrete structures were used, which mainly consisted of aggregates with an attached mortar. The size fraction was 4–8 mm (4–8 R) with a fineness modulus of 6.95. Finally, the fine aggregate was natural sand with a maximum aggregate size of 2 mm (0–2 N) and a fineness modulus of 4.70. Limestone powder (LP) was used as a microfiller [35]. Table 1 shows the basic properties of the aggregates used in this study. Figure 1 illustrates the composition of the recycled coarse aggregates according to EN 933-11. The recycled aggregate consisted mainly of crushed cement concrete (Rc = 88.5%). Based on these results, they can be classified as recycled coarse aggregates (RCAs) from concrete demolition waste. Five different concrete compositions were chosen for this study, differing in the degree of replacement of natural aggregate (4–8 N) with recycled aggregate (4–8 R) from 0% to 100%. A constant resin/microfiller ratio of 1.0 was assumed. The choice of a ratio equal to 1.0 is driven by the desire to achieve a composite with appropriate workability while simultaneously minimizing the use of resin. Minimizing the use of resin is due to it being the most expensive and least environmentally friendly component of the proposed composite. However, the limitation in minimizing its usage lies in achieving the appropriate workability and ensuring that the aggregate grains are coated with an adequate layer of micro-mortar. The resin content was constant, 300 kg. The recycled aggregate was washed with a 0.125 mm sieve and dried in a drying oven at 100 °C for 48 h. The mass compositions of the composites per cubic meter are listed in Table 2.

**Table 1:** Basic properties of the aggregate. LP: limestone powder, 0–2 N natural sand, 2–4 N, 4–8 N natural gravel, 4–8 R recycled aggregate.

Property	LP	0–2 N	2–4 N	4–8 N	4–8 R
Density (EN 1097-6 [36]), g/cm^3^	2.71	2.54	2.64	2.67	2.53
Density in oven-dry conditions (EN 1097-6 [36]), g/cm^3^	-	2.13	2.46	2.60	2.25
Water absorption (EN 1097-6 [36]), %	-	7.5	2.8	1.2	4.8
Los Angeles Abrasion (EN 1097-2 [37]), %	-	-	27.0	25.0	75.0
Fines percentage (EN 933-1 [38]), %	100	1.0	0.1	0.1	0.9

**Table 2:** Composites composition.

In Weight	R00	R10	R20	R30	R40
Synthetic vinyl-ester resin, kg	300	300	300	300	300
Limestone powder, kg	300	300	300	300	300
Natural sand (0–2 N), kg	600	600	600	600	600
Gravel (2–4 N), kg	300	300	300	300	300
Gravel (4–8 N), kg	600	450	300	150	0
Recycled aggregate (4–8 R), kg	0	150	300	450	600
RA in total aggregate, %	0	10	20	30	40
Gravel replacement, %	0	25	50	75	100

The consistency of the mixture was evaluated according to the procedure for measuring the plasticity of construction mortars (according to the PN-EN 1015-3 standard [39]) immediately after mixing the ingredients. A truncated cone (bottom diameter of 100 mm, top diameter of 70 mm, and height of 60 mm) was shaped on the flow table. Therefore, the shaped fresh composite was subjected to 15 generative shakes by lifting and dropping the measuring table to a height of 10 mm at a rate of 1 per second). The diameter of the resulting flow is measured. To test the flexural strength of the composites, three rectangular specimens measuring 40 mm × 40 mm × 160 mm were prepared for each composition, and tested according to the EN 196-1 [40] standard using a three-point loading method. The rate of increase in flexural strength was 50 N/s. For compressive strength testing, six specimens were prepared for each composition, with a compressive area of 1600 mm^2^, and were also tested according to the EN 196-1 [40] standard. The rate of increase in compressive load was 2400 N/s. Both flexural and compressive strengths were determined with an accuracy of 0.1 MPa.

Statistical analysis of the obtained results of the flexural and compressive strength tests was performed to determine whether the differences between the groups were statistically significant. RStudio 2023.12 software was used as a tool for analysis, which provided accurate results. RStudio is an integrated development environment used to facilitate and improve work with R, a programming language, and environment for data analysis and statistics. It allows for data manipulation, statistical analysis, graph creation, data modelling, and many other tasks related to data analysis [41]. The analysis used the analysis of variance (ANOVA)—a statistical technique for comparing the mean values between groups to determine whether there are statistically significant differences between these groups [42]. In the case of the analysis of variance (ANOVA), the value of the F-statistic is determined, which indicates the ratio of the variance between groups to the variance within groups. The higher the value of the F-statistic, the greater the difference between the mean values between groups compared with the variance within groups. Then, the *p*-value was determined, a value indicating the statistical significance of the differences between the groups of results. If this value is smaller than the significance level alpha, which was set as a typical coefficient that is commonly used in materials engineering at 0.05 [43], this means that there is a statistically significant difference between the groups of results. If the difference between any pair of results turned out to be significant, the pairwise *t*-test function was used to perform multiple tests on different pairs of groups of results to compare their means. In the case of multiple comparisons of results from several groups, it is necessary to adjust the significance level for the tests to avoid a very high chance of making a type I error—an error consisting of rejecting a true null hypothesis [44]. The pairwise *t*-test function performs a correction of *p*-values using one of several methods, such as the Bonferroni method [45], to ensure an appropriately low significance level for multiple tests and avoid a type I error.

Microstructure observations were carried out using a scanning electron microscope (SEM) produced by Zeiss, model Sigma 500 VP (Carl Zeiss Microscopy GmbH, Köln, Germany). Backscattered electron (BSE) images were obtained. The phase compositions and mapping were analyzed using an energy-dispersive X-ray spectroscopy (EDX) detector produced by the Oxford model Ultim Max 40.

**Figure 2:**
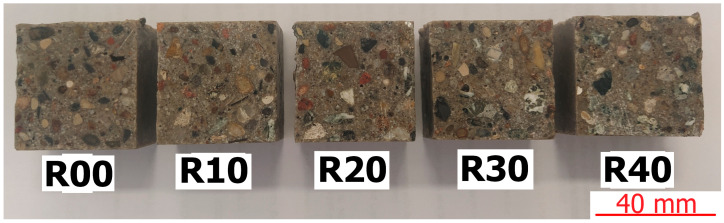
Cut and pre-polished sections of tested polymer composites.

Five types of polymer composite samples (R00, R10, R20, R30, and R40) were used as test materials. From each type of received bar of material with dimensions of 40 × 40 × 160 mm^3^, a thick slice from the middle section was cut perpendicular to the trowelling surface. The surfaces were ground and polished in this manner. The cut and pre-polished sections of the polymer composites are shown in Figure 2—the scale bar has been placed in the lower right corner of the figure.

**Figure 3:**
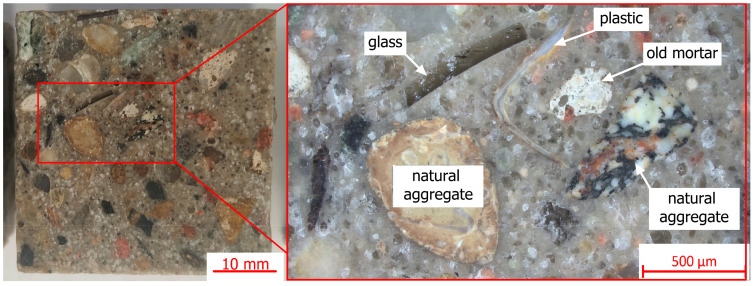
Example of various types of particles in R10 composite.

Figure 3 presents various types of waste materials present in the recycled aggregates used to prepare the test samples—the scale bar has been placed in the lower right corner of the figure. Observations were performed using a stereoscopic optical microscope (ZEISS Stemi 508 (Jena, Germany)).

Optical microscopy examinations of the prepared samples showed various types of materials applied to polymer concrete and recycled aggregate. Apart from the natural aggregates covered by old mortar or grains of old mortar, glass and plastic particles were also present.

For the SEM examinations, a selected region from the pre-polished sections was cut with surface dimensions of approximately 20 mm × 20 mm. After cutting, the samples were dried in an oven and placed in epoxy resin for better handling during the polishing process. Microscopic sections were prepared in the same way as in previously published papers [46]—the final step of preparing the samples after polishing their surface was gold evaporation before examining them under a microscope.

## 3. Results

The consistency tests revealed that all the compositions had a similar composite spread of (180 ± 5) mm every time. This indicates that the recycled aggregate did not change the basic rheological properties of the mixtures (Table 3). The replacement of natural aggregate with recycled aggregate, regardless of the replacement level, did not affect the workability of the mix. During mixing and placing into molds, no reduction in workability was observed. Perhaps the use of a lower viscosity resin, allowing it to penetrate the porous recycled aggregate particles during mixing, could potentially worsen the workability of the mix. The apparent density of the composites ranged from 2.12 g/cm^3^ to 2.18 g/cm^3^. The apparent density of the composites changes only slightly with the increasing recycled aggregate content—the differences are within the standard deviation limits (Table 3). This relationship was maintained despite the significant difference in density between recycled aggregate grains and natural aggregate grains. The lower density of recycled aggregate is due to the presence of porous cement paste grains. The lack of a significant difference in the apparent density of the polymer composite suggests that the resin penetrated the pores of the recycled aggregate and filled them. For all the analyzed composites, a significant flexural strength of approximately 21 MPa was achieved. The use of recycled aggregate did not significantly affect the flexural strength of the composites (Table 3). The tests also yielded high compressive strength results of around 90 MPa. No increase in the standard deviations of the results was observed with the increasing proportion of recycled aggregate in the mixtures, which could have occurred due to the heterogeneity of the recycled aggregate used. The presence of locally weaker grains did not affect the greater variability in the test results of the samples (Table 3).

**Figure 4:**
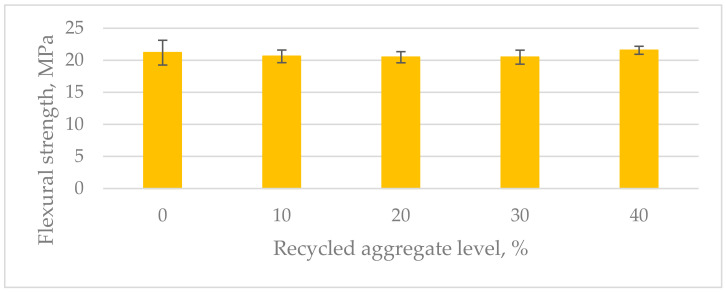
Flexural strength of tested composites.

**Figure 5:**
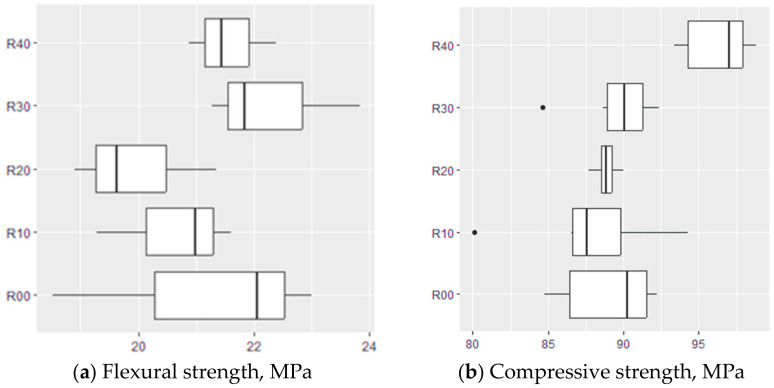
Boxplot of strength results for each group: (**a**) Flexural strength, (**b**) Compressive strength.

**Figure 6:**
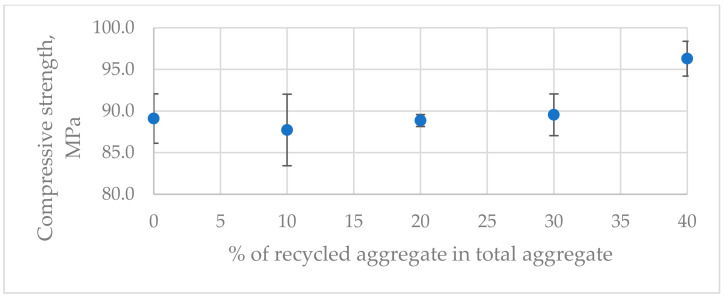
Compressive strength of tested composites.

The average flexural strength was between 20.5 and 21.5 MPa (Figure 4). The differences among the average values for the different compositions were within the range of the standard deviations of the results (Figure 5). The average compressive strength results were between 88.5 and 96.5 MPa (Figure 6). The differences among the average values for the different compositions were also within the range of the standard deviations of the results. The highest compressive strength value of 96.5 MPa was achieved by the samples with the highest recycled aggregate content (Figure 5). This was surprising because the recycled aggregate itself was weaker than the natural aggregate. This could be explained by the fact that the resin binder filled the hardened cement slurry, making it stronger and improving the resin–aggregate transition zone. This hypothesis was also supported by the breaking of the specimens during the flexural and compressive tests. The fracture surface passed through the hardened resin micropaste as well as through the recycled aggregate. No destruction was observed in the zone where the resin bonded with the recycled aggregate, as is often the case with polymer concrete containing only natural aggregates.

Microstructural SEM examinations focused mainly on coarse aggregates and the transition zone between the resin matrix and grains. These types of areas were analyzed by choosing various types of recycled aggregates (RAs), and the results were compared to a reference sample (R00) containing only natural coarse aggregate.

For the obtained results of flexural and compressive strengths, statistical analyses were carried out to verify the existence of differences between groups—composites with different contents of recycled aggregate.

Explanation of table abbreviations: Df—degrees of freedom. This observation number independently contributes to the estimation of the mean for each group or the mean for the residuals. The higher the number of degrees of freedom, the greater the precision of the estimator. Sum Sq— Sum of squares. This is a measure of the total variability of the sample. Mean Sq—mean squares. This is the average value of the square of the differences between the values of the groups. F value—This is the test statistic used to evaluate whether the differences between groups or between residuals are statistically significant. The higher this value, the more likely it was that the differences between the groups were statistically significant. Pr(>F)—*p*-value. The smaller this value, the less likely it is that the differences between groups are due to chance, and more likely that these differences are statistically significant.

In the study of flexural strength, it was assumed that if the *p*-value was less than 0.05, then the differences between the groups were not due to chance. With 4 degrees of freedom, the sum of squares is 9.653, the mean square is 2.4133, and the F value is 1.0995. The *p*-value of 0.4085 suggests that the differences between groups are not statistically significant, as this value is relatively high. With 10 degrees of freedom, the sum of squares is 21.950, and the mean square is 2.1949. The F value is not applicable for residuals (Table 4). From the results obtained using RStudio, the *p*-value for the bending strength results was 0.4085. This means that this value is higher than the significance level alpha of 0.05, and there is no statistically significant difference between the bending strength results.

In the study of compressive strength, with 4 degrees of freedom, the sum of squares is 279.64, the mean square is 69.909, and the F value is 7.5803. The *p*-value is 0.0003829, which is very small, indicating that the differences between groups are statistically significant. In the case of residuals, with 25 degrees of freedom, the sum of squares is 230.56 and the mean square is 9.222. The F value is not applicable for residuals. In the case of compressive strength results, there was a statistically significant difference between the results; the *p*-value was 0.0003829, which was smaller than the significance level alpha of 0.05 (Table 5).

For further analysis, the pairwise *t*-test function mentioned in point 5.5 was used with the correction of *p*-values by the Bonferroni method and a further assumption of a significance level equal to 0.05. The results obtained using this function are presented in Table 6.

It was observed that as the recycled aggregate content in the mixture increased, the compressive strength also increased. This trend is mainly driven by the complete replacement of natural aggregate with recycled aggregate, indicating a qualitative rather than a quantitative change in the mixture. This was confirmed by statistical analyses.

Reference polymer concrete

**Figure 7:**
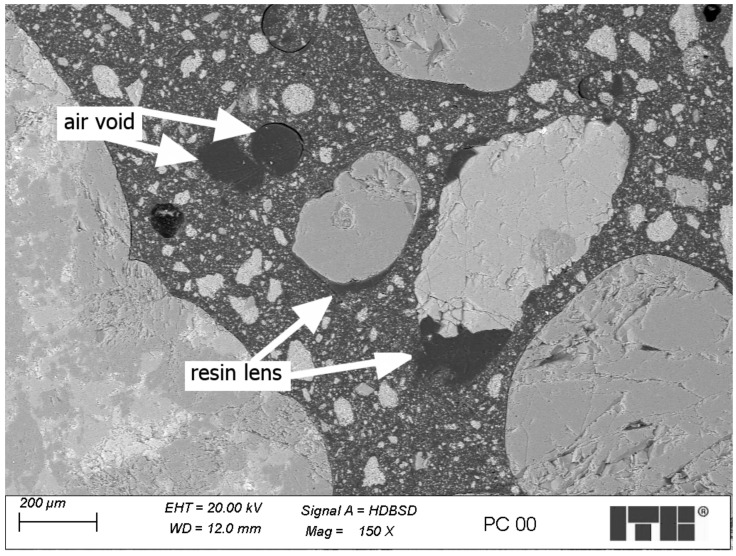
Microstructure of reference polymer concrete—sample R00.

The reference PC contains coarse aggregates with relatively low-porosity grains which do not absorb much resin in the grain surface zone. The polymer matrix contains a small number of air voids. The surfaces of most grains of the coarse aggregates were covered with resin. The filler grains were evenly distributed in the matrix. Filler-free resin lenses were observed under some coarse aggregate grains, as shown in Figure 7—the scale bar has been placed in the lower right corner of the figure. This might be caused by the relatively low viscosity of the resin and the low homogeneity due to the ineffective mixing and compacting process.

Recycled Aggregate (RA) polymer concrete

**Figure 8:**
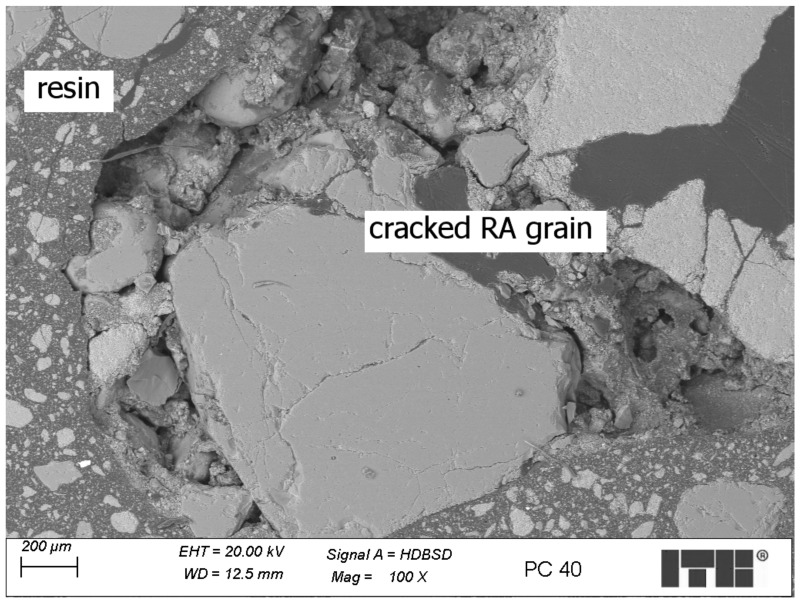
Weak and cracked grain of RA surrounded by the resin matrix—sample R40.

Analysis of the microstructure of polymer concrete containing RA revealed some weak and cracked grains of RA which were surrounded and enhanced by the resin matrix. However, the resin did not fully fill the cracks, and the strengthening of such grains may not be fully effective. An example of such a grain is shown in Figure 8—the scale bar has been placed in the lower right corner of the figure.

**Figure 9:**
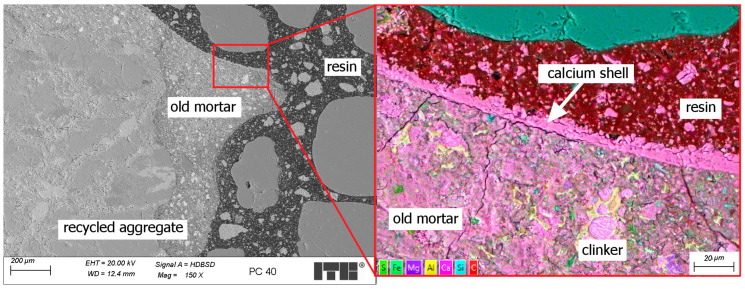
Example of double-layer shell formed on the surface of porous RA grain—sample R40.

The most important SEM observation is that the porous grains of RA had a double-layer shell—one inside the grain formed by saturation with resin, and the other on the grain surface formed by the calcite filler. An example of such a structure is shown in Figure 9—the scale bar has been placed in the lower right corner of the figure.

The mechanism of forming the inner shell is formed by the absorption of resin by the porous grain. The outer shell is formed by increasing the concentration of filler in the areas near the surface of the porous grains. This phenomenon can be described as the sieving effect. The porous surface of the recycled aggregate grains acted similar to a sieve, allowing the resin to pass into the grain while retaining the microfiller on the surface of the grains. This resulted in the concentration of the microfiller on the surface of the recycled aggregate grains. The above phenomenon was not observed on grains with low porosity, for example, natural aggregates, glass, or plastic particles which do not absorb the resin.

## 4. Discussion and Conclusions

Due to the fact that workability does not change with increasing proportions of recycled aggregate, it was feasible to entirely replace natural coarse aggregate with recycled aggregate. The flexural strength remains consistent with the substitution of natural aggregate by recycled aggregate, maintaining a value of approximately 22 MPa. Similarly, the compressive strength remains largely unchanged when partially replacing natural coarse aggregate with recycled aggregate, with variations confined within the standard deviation and averaging around 90 MPa. Notably, a significant increase in compressive strength of about 6% is observed when natural coarse aggregate is completely substituted with recycled aggregate. This is not obvious considering the inferior mechanical properties of RA compared to those of most natural aggregates. However, during the SEM examinations, it was found that the surface of the porous RA grains absorbed the resin and formed a double-layer shell with the calcium filler, which might increase the mechanical properties of the polymer concrete. The investigations showed that RA might be a valuable material for PC, especially when it contains porous grains with rather low mechanical properties. These RAs may not be effective in other applications, such as Portland cement composites, because of their poor mechanical properties. The findings of this study suggest new, promising directions for future research. Resin with lower viscosity can enhance crack filling in the grains of recycled aggregate (RA), potentially increasing the compressive strength of the composite. Additionally, such a resin may penetrate deeper into the porous RA grains, further strengthening them.

## Figures and Tables

**Table 3:** Flow, apparent density, flexural strength, and compressive strength of the composites.

Composition	Flow, mm	Apparent Density ρ, g/cm^3^	Flexural Strength f_t_, MPa	Compressive Strength fc, MPa
R00	180 ± 20	2.18 ± 0.03	21.2 ± 2.4	89.1 ± 3.3
R10	180 ± 20	2.15 ± 0.02	20.6 ± 1.2	87.7 ± 4.7
R20	180 ± 20	2.12 ± 0.05	20.0 ± 1.3	88.9 ± 0.8
R30	180 ± 20	2.14 ± 0.01	22.3 ± 1.3	89.6 ± 2.7
R40	180 ± 20	2.17 ± 0.02	21.6 ± 0.8	96.3 ± 2.3

**Table 4:** Analysis of variance of flexural strength results (RStudio program).

	Df	Sum Sq	Mean Sq	F Value	Pr(>F)
Groups	4	9.653	2.4133	1.0995	0.4085
Residuals	10	21.950	2.1949		

**Table 5:** Analysis of variance of compressive strength results (RStudio program).

	Df	Sum Sq	Mean Sq	F Value	Pr(>F)
Groups	4	279.64	69.909	7.5803	0.0003829
Residuals	25	230.56	9.222		

**Table 6:** *T*-tests for pairs with Bonferroni correction for multiple comparisons.

	R00	R10	R20	R30
R10	1.0000	-	-	-
R20	1.0000	1.0000	-	-
R30	1.0000	1.0000	1.0000	-
R40	0.0038	0.0005	0.0027	0.0073

## Data Availability

The original contributions presented in the study are included in the article, further inquiries can be directed to the author.

## References

[1] Blomsma F., Brennan G. (2017). The Emergence of Circular Economy: A New Framing Around Prolonging Resource Productivity. J. Ind. Ecol..

[2] Anwar M.K., Shah S.A., Alhazmi H. (2021). Recycling and Utilization of Polymers for Road Construction Projects: An Application of the Circular Economy Concept. Polymers.

[3] Colangelo F., Navarro T.G., Farina I., Petrillo A. (2020). Comparative LCA of Concrete with Recycled Aggregates: A Circular Economy Mindset in Europe. Int. J. Life Cycle Assess..

[4] Kępniak M., Załęgowski K., Woyciechowski P., Pawłowski J., Nurczyński J. (2022). Feasibility of Using Biochar as an Eco-Friendly Microfiller in Polymer Concretes. Polymers.

[5] International Solid Waste Association The Future of the Waste Management Sector. Trends, Opportunities and Challenges for Decade. https://www.iswa.org/.

[6] Nuaklong P., Sata V., Chindaprasirt P. (2016). Influence of Recycled Aggregate on Fly Ash Geopolymer Concrete Properties. J. Clean. Prod..

[7] Selva Ganesh M., Jagadeesh P. (2022). Assessment of Usage of Manufactured Sand and Recycled Aggregate as Sustainable Concrete: A Review. Mater. Today Proc..

[8] Jiang Y., Li B., Liu S., He J., Hernandez A.G. (2022). Role of Recycled Concrete Powder as Sand Replacement in the Properties of Cement Mortar. J. Clean. Prod..

[9] Garbacz A., Sokołowska J.J. (2013). Concrete-like Polymer Composites with Fly Ashes—Comparative Study. Constr. Build. Mater..

[10] Barbuta M., Bucur R.D., Cimpeanu S.M., Paraschiv G., Bucur D., Pilipavičius V. (2015). Wastes in Building Materials Industry.

[11] Agavriloaie L., Oprea S., Barbuta M., Luca F. (2012). Characterisation of Polymer Concrete with Epoxy Polyurethane Acryl Matrix. Constr. Build. Mater..

[12] Kaya A., Kar F. (2016). Properties of Concrete Containing Waste Expanded Polystyrene and Natural Resin. Constr. Build. Mater..

[13] Czarnecki L., Łukowski P., Nejman R. (1996). The Statistical Evaluation of Epoxy Concrete Heterogeneity. Cem. Concr. Compos..

[14] Ribeiro M.S.S., Tavares C.M.L., Ferreira A.J.M. (2002). Chemical Resistance of Epoxy and Polyester Polymer Concrete to Acids and Salts. J. Polym. Eng..

[15] Kȩpniak M., Woyciechowski P., Franus W. (2017). Chemical and Physical Properties of Limestone Powder as a Potential Microfiller of Polymer Composites. Arch. Civ. Eng..

[16] Łukowski P., Sokołowska J., Kępniak M. (2014). The Introductory Evaluation of Possibility of Using Waste Perlite Powder in Building Polymer Composites. Bud. Archit..

[17] Bulut H.A., Şahin R. (2017). A Study on Mechanical Properties of Polymer Concrete Containing Electronic Plastic Waste. Compos. Struct..

[18] Tonet K.G., Gorninski J.P. (2013). Polymer Concrete with Recycled PET: The Influence of the Addition of Industrial Waste on Flammability. Constr. Build. Mater..

[19] Woyciechowski P., Kępniak M., Pawłowski J. (2022). Methodology for measuring the carbonation depth of concrete–standard and non-standard aspects. Struct. Environ..

[20] Tang B., Fan M., Yang Z., Sun Y., Yuan L. (2023). A Comparison Study of Aggregate Carbonation and Concrete Carbonation for the Enhancement of Recycled Aggregate Pervious Concrete. Constr. Build. Mater..

[21] Qin W., Fan X., Jiang X. (2024). CO2-Accelerated Carbonation Modification for Recycled Coarse Aggregate with Various Original Concrete Strengths and Coarse Aggregate Sizes. Materials.

[22] Raman J.V.M., Ramasamy V. (2021). Various Treatment Techniques Involved to Enhance the Recycled Coarse Aggregate in Concrete: A Review. Mater. Today Proc..

[23] Spaeth V., Djerbi Tegguer A. (2013). Improvement of Recycled Concrete Aggregate Properties by Polymer Treatments. Int. J. Sustain. Built Environ..

[24] Li L., Liu K., Chen B., Wang R. (2022). Effect of Cyclic Curing Conditions on the Tensile Bond Strength between the Polymer Modified Mortar and the Tile. Case Stud. Constr. Mater..

[25] Liu J., Ma K., Shen J., Zhu J., Long G., Xie Y., Liu B. (2023). Influence of Recycled Concrete Aggregate Enhancement Methods on the Change of Microstructure of ITZs in Recycled Aggregate Concrete. Constr. Build. Mater..

[26] Misra A.K., Kalra M., Bansal S. (2017). Influence of Polymer Treatment on Strength and Water Absorption Capacity of Recycled Aggregate Concrete. Int. J. Sustain. Build. Technol. Urban Dev..

[27] Velardo P., Sáez del Bosque I.F., Sánchez de Rojas M.I., De Belie N., Medina C. (2022). Durability of Concrete Bearing Polymer-Treated Mixed Recycled Aggregate. Constr. Build. Mater..

[28] Spaeth V., Djerbi Tegguer A. (2013). Polymer Based Treatments Applied on Recycled Concrete Aggregates. Adv. Mater. Res..

[29] Wang Y., Zheng J., You F. (2021). Review on Enhancement Methods of Recycled Aggregate. Cailiao Daobao/Mater. Rep..

[30] Mercuri M., Vailati M., Gregori A. (2023). Lime-Based Mortar Reinforced with Randomly Oriented Polyvinyl-Alcohol (PVA) Fibers for Strengthening Historical Masonry Structures. Dev. Built Environ..

[31] Angiolilli M., Gregori A., Vailati M. (2020). Lime-Based Mortar Reinforced by Randomly Oriented Short Fibers for the Retrofitting of the Historical Masonry Structure. Materials.

[32] Li L., Wang R., Lu Q. (2018). Influence of Polymer Latex on the Setting Time, Mechanical Properties and Durability of Calcium Sulfoaluminate Cement Mortar. Constr. Build. Mater..

[33] Polimal. https://www.polimal.com.pl/.

[34] Darimex. https://www.kopalniadarimex.pl/.

[35] EGM. https://egm.pl/.

[36] (2022). Tests for Mechanical and Physical Properties of Aggregates—Part 6: Determination of Particle Density and Water Absorption.

[37] (2020). Tests for Mechanical and Physical Properties of Aggregates—Part 2: Methods for the Determination of Resistance to Fragmentation.

[38] (2012). Methods of Test for Dense Shaped Refractory Products—Part 1: Determination of Bulk Density, Apparent Porosity and True Porosity.

[39] (1999). Methods of Test for Mortar for Masonry—Part 3: Determination of Consistence of Fresh Mortar (by Flow Table).

[40] (2016). Methods of Testing Cement Part 1: Determination of Strength.

[41] Nisbet R., Elder J., Miner G. (2009). Chapter 6—Accessory Tools for Doing Data Mining.

[42] Ben Taher M.A., Pelay U., Russeil S., Bougeard D. (2023). A Novel Design to Optimize the Optical Performances of Parabolic Trough Collector Using Taguchi, ANOVA and Grey Relational Analysis Methods. Renew. Energy.

[43] Chén O.Y., Bodelet J.S., Saraiva R.G., Phan H., Di J., Nagels G., Schwantje T., Cao H., Gou J., Reinen J.M. (2023). The Roles, Challenges, and Merits of the p Value. Patterns.

[44] Liu W., Jia Y.-X. (2023). Comparison of Type I Error and Statistical Power between State Trace Analysis and Analysis of Variance. J. Math. Psychol..

[45] Lesack K., Naugler C. (2011). An Open-Source Software Program for Performing Bonferroni and Related Corrections for Multiple Comparisons. J. Pathol. Inform..

[46] Chyliński F., Michalik A., Kozicki M. (2022). Effectiveness of Curing Compounds for Concrete. Materials.

